# E‑Cigarettes Generate Gas Phase Ions

**DOI:** 10.1021/envhealth.5c00788

**Published:** 2026-04-20

**Authors:** Nicole C. Auvil, Mark E. Bier

**Affiliations:** Department of Chemistry, 6612 Carnegie Mellon University, Pittsburgh, Pennsylvania 15213, United States

**Keywords:** e-cigarette, vape, smoke, aerosol, atmospheric pressure ionization, thermospray ionization, mass spectrometry, gas phase ion inhalation

## Abstract

Mass spectrometry (MS) evidence shows that electronic cigarettes (e-cigs) can directly emit gas phase ions at levels orders of magnitude greater than detected from typical ambient air or smoke from the mouthpiece of a traditional tobacco cigarette (t-cig). These ions, generated upon atomization of the e-liquid into an e-cig aerosol, are presumably inhaled by e-cig users. Emissions from disposable e-cigs were sampled at atmospheric pressure directly into a mass spectrometer without a conventional ion source. All four e-cigs produced mass spectra with high ion intensities, indicating that their aerosols were ion-rich upon exiting the mouthpieces. While multiple ionization pathways may contribute, atmospheric pressure thermospray ionization (APTSI) appeared to be the dominant pathway. The e-cig self-ionization process provides a direct method of aerosol analysis by mass spectrometry (MS) and eliminates the need for precollection and conventional MS ion sources. This finding has implications for e-cig user exposure that warrant further mechanistic and toxicological study.

## Graphical abstract

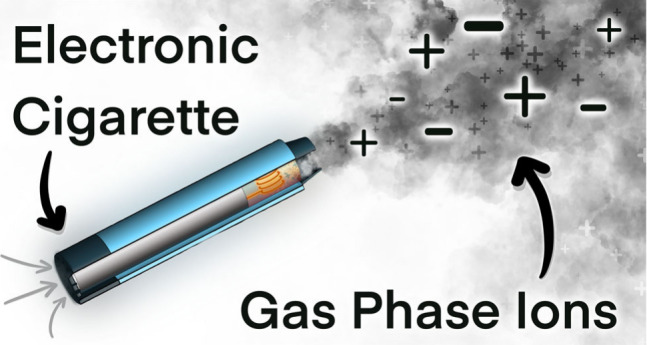

## Introduction

Electronic cigarettes, also known as e-cigs or vaping devices, were developed as an oral nicotine delivery alternative to traditional tobacco cigarettes (t-cigs).^1^ E-cig manufacturers may produce different designs, but the devices typically contain the same basic components: a battery, an e-liquid reservoir, a wicking material, and a heated filament. A user turns on the device by a power switch or by triggering a lower pressure sensor by suction through the mouthpiece. An electrical current then passes through the filament in contact with the e-liquid-soaked wick and rapidly reaches temperatures high enough to atomize the e-liquid into a dense aerosol.^2^ The aerosol is then inhaled by the user.^3^ The user not only inhales a dose of e-liquid ingredients, but also products of their thermal decomposition and other chemical reactions.^3–7^

Because e-cigs forego the combustion process essential to tobacco based cigarettes, vaping may be perceived as a safer alternative to and even a method to wean oneself from smoking.^5^ However, e-cigs have been shown to adversely impact indoor air quality and human health.^8,9^ E-cig use has been associated with ion transport dysfunction in airway epithelial cells,^6^ increased lung cancer risk in rats,^5^ and potentially fatal pneumonia-like respiratory injury.^10^ Just one session of e-cig use has been shown to increase oxidative stress levels in healthy people.^11^ Despite their pressing nature, the mechanistic pathways of many e-cig related health ailments remain unexplained.^12^

Hybrid techniques such as gas chromatography mass spectrometry (GC/MS) and liquid chromatography MS (LC/MS) have widely been used to identify and quantify chemicals in e-liquids.^13^ However, the main e-liquid components, including propylene glycol (PG), vegetable glycerin (VG), nicotine (N), and flavorants, are known to thermally degrade and undergo reactions with radicals during the vaping process. These reactions result in the formation of biologically harmful species such as carbonyls, organic free radicals, reactive oxygen species (ROS), and reactive nitrogen species (RNS).^14–18^ Since the chemical composition of an e-liquid is not necessarily equivalent to that of the aerosol it produces, it is imperative to directly determine the composition of e-cig aerosol itself in order to identify the chemicals to which e-cig users and bystanders are exposed.

Aerosols can require specialized techniques for gas phase molecular analysis using standard MS. One solution is to precollect e-cig aerosol and subsequently use standard MS methods to analyze the concentrate. These preanalysis treatments, such as vapor trapping and solid phase microextraction (SPME), can be costly in terms of time and materials and may lead to sample losses or unwanted chemical reactions.^7,19^ For a more direct analysis, atmospheric pressure MS e-cig sampling devices have been used. Commercial proton transfer reaction MS (PTR-MS),^20^ secondary electrospray ionization MS (SESI-MS),^4^ and direct analysis in real time MS (DART-MS)^7,19^ have been modified to directly analyze e-cig aerosol in the gas phase in real time as it is produced. These interfaces desolvate the aerosol and, in the process, ionize the molecules for mass analysis.

However, a limitation of these conventional ion sources is that they may generate chemical species that are not present in the original aerosol.^21^ The species can become confounded, making it difficult to accurately determine their origin. In this study we report the observation of gas phase ion formation by e-cigs, which allowed for direct analysis of e-cig emissions without a conventional ion source on the mass spectrometer.^22,23^ Nanoelectrode atmospheric pressure chemical ionization (nAPCI) and/or nanoelectrode electrospray ionization (nESI) were used as the ion source in control experiments.^24,25^ The e-cig self-ionization discovery highlights a previously unrecognized pathway for chemical exposure in e-cig users: gas phase ion inhalation.

## Materials and Methods

Four different nicotine e-cigs purchased new locally were analyzed in this study. They were all disposable and suction activated. E-cig 1 was SWFT PRO brand (cool mint flavor, 50 mg/mL nicotine, 2000 puffs, 6 mL, Shenzhen Technology Co., China). E-cig 2 was FUNKY REPUBLIC brand (Miami mint flavor, 40 mg/mL nicotine, 3000 puffs, 5 mL, batch no. FP023816, Shenzhen Technology Co., China). E-cig 3 was the PUFF PLUS brand (lush flavor, 50 mg/mL nicotine, 800 puffs, 3.5 mL, PVG Inc., USA). E-cig 4 was the EBCREATE brand (banana cake flavor, 50 mg/mL nicotine, 5000 puffs, 9.5 mL, batch no. EBCP026950, Shenzhen Technology Co., China). Manufacturers did not provide a PG:VG ratio or type of nicotine salt, but these are known to be constituents of disposable e-cig e-liquids. One t-cig was analyzed in this study, Marlboro brand (red, filtered, batch no. V335 U31B5, Philip Morris USA, USA).

Aerosols produced by the t-cig and four e-cigs were analyzed with two different atmospheric pressure interface mass spectrometers. An LCQ Deca XP Plus mass spectrometer (ThermoFisher Scientific, San Jose, CA, USA) was used to collect the “puffs over time” data in Figure 2. The settings were as follows: ion transfer tube temperature: 250 °C; ion transfer tube voltage: 13 V; tube lens: −30 V; microscans: 5, scan range: 50–2000 *m*/*z*; positive ion mode; normal scan mode. For improved detection, an LTQ XL mass spectrometer (ThermoFisher Scientific, San Jose, CA, USA) with linear ion trap technology incorporating a larger trapping volume was used for the additional experiments.^26^ The settings were as follows: ion transfer tube temperature: 200 °C, ion transfer tube voltage: 50 V, tube lens: 100 V, microscans: 5, maximum ion inject time: 10 ms, scan range: 100–2000 *m*/*z*, positive and negative ion mode, normal scan mode.

The e-cig was perpendicularly connected via the mouthpiece to the MS inlet with a polyether ether ketone (PEEK) adapter assembly (1.59 mm i.d. with 1.25 mm thru hole, IDEX Health & Science, USA). The junction between the mouthpiece and the assembly was bridged with stainless steel tubing (1.59 mm o.d. x 1.17 mm i.d., IDEX Health & Science, USA), ethylene tetrafluoroethylene (ETFE) tubing (3.18 mm o.d. x 1.59 mm i.d. IDEX Health & Science, USA), and silicone tubing (MODDIY, China) of varying size to form a tight seal around the mouthpiece of each cigarette. A vacuum pump (1 L/min, SKC Airchek Sampler) was connected to the assembly directly opposite the e-cig. The remaining open port of the assembly was opened or closed using a solid threaded HPLC fitting. When open, the mass spectrometer sampled the ambient laboratory air. When closed, a vacuum was pulled on the e-cig mouthpiece, simulating inhalation and activating aerosol production. The mass spectrometer sampled cigarette aerosol perpendicularly out of the main aerosol stream. No additional ion source was added for these experiments. For control experiments with a conventional ion source, nAPCI was used.^24^ A nanosharp nAPCI corona needle was added to a fifth port in the assembly and was attached to a high voltage power supply (Model 205B-10R, Bertan Associates Inc., USA) to generate corona discharge. Under the aerosolization of the e-liquid, however, microdroplet loading onto the enclosed nAPCI needle tip would be expected to stop the corona discharge but then start the process of nESI from the same electrode tip.

## Results and Discussion

### Cigarette–Mass Spectrometer Interface

Four different disposable nicotine e-cigs and one t-cig were analyzed using an atmospheric pressure interface MS. The mouthpiece of each e-cig was connected perpendicularly to the MS inlet with a 5-port polyether ether ketone (PEEK) assembly in the orientation depicted in Figure 1. Tubing size was selected to form an airtight seal around the mouthpiece of each e-cig. To simulate a “puff”, a SKC vacuum pump was connected to the assembly parallel to the e-cig and set to 1 L/min which was sufficient to activate aerosol production and in agreement with reported human e-cig puffing behavior.^27^ A remaining open port (pointing out-of-page in Figure 1) was opened or closed as needed using a solid threaded HPLC fitting. When the port was open, there was negligible suction on the mouthpiece; the MS inlet primarily sampled ambient lab air as a puff. When the port was closed, the suction-activated circuit turned the filament on, and the e-cig produced an aerosol combined with lab air. These emissions were pulled through the e-cig device and sampled into the MS interface perpendicular to the main aerosol stream. Since the e-cig self-ionized the e-liquid directly, no additional ion source was used in this setup. For control experiments using conventional ion sources, nAPCI and/or nESI were used.^24,25^ The dual use emitter needle was placed in the fifth assembly port, coaxial to the MS inlet, and was attached to a high voltage power supply to generate either an ionizing corona discharge or, if the electrode was loaded with e-liquid aerosol condensation, electrosprayed microdroplets. This needle electrode dual mode ionization has previously been observed in our lab.^200^ Further studies are needed to deconvolute these two ionization processes and their interactions with the e-cig aerosol.

**Figure 1:**
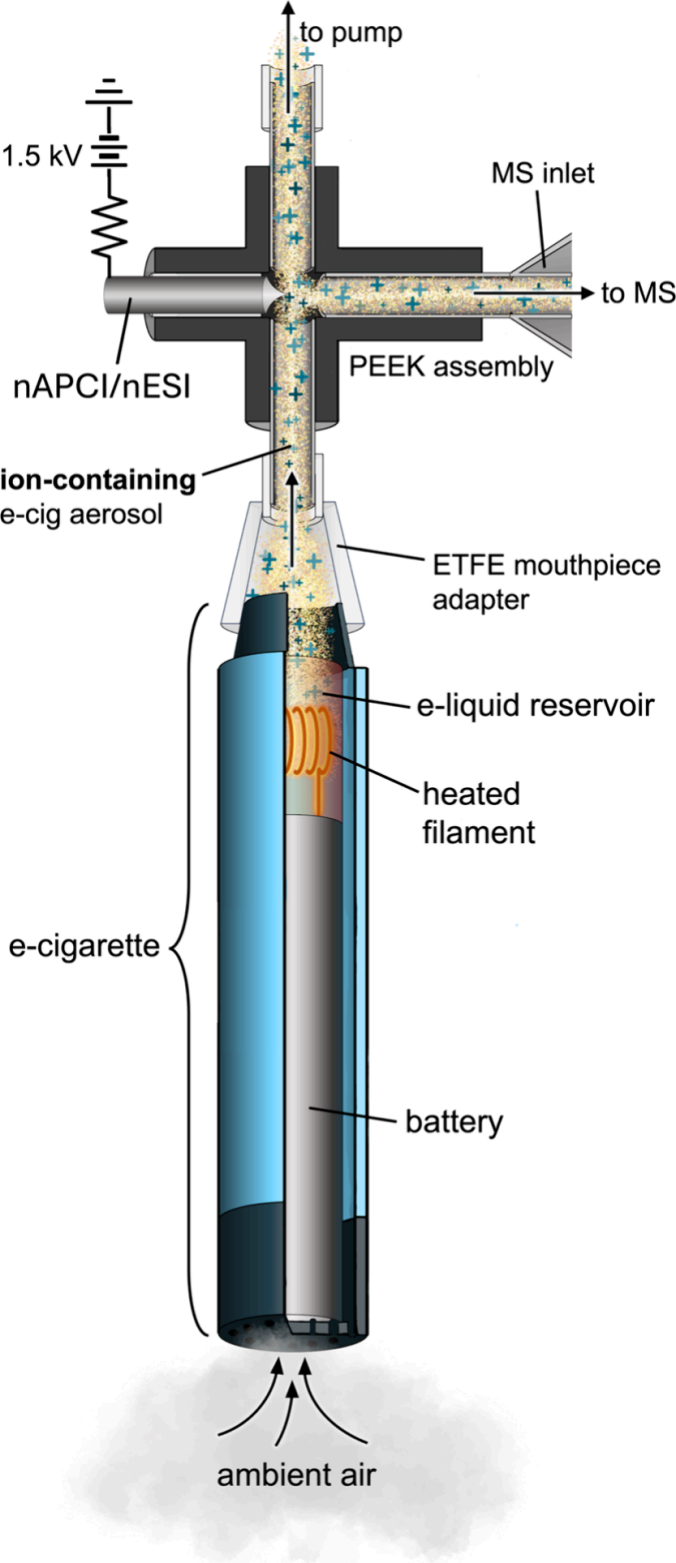
Cross-sectional diagram of the e-cig aerosol analysis interface. The emission needle was operated in APCI mode until the e-cig turned on, at which time the needle is believed to become an nESI emitter. The needle was removed, and its port was closed for experiments without a conventional ion source. A fifth port pointing out-of-page, not pictured, was opened or closed as needed by using a solid threaded HPLC fitting. Simplified visualization of e-cig internals. Not to scale.

### Detecting Ions Directly from E-Cigs

Figure 2 contains data collected using MS over short bursts of aerosol production known as puffs from e-cig 1 without a conventional ion source. The extracted ion chromatograms show that MS ion intensity was temporally correlated with puffing, indicating that detected ions originated from the e-cig during aerosol production. Puffs, which were ∼ 5 s in duration and occurred every ∼ 30 s, remained consistent in overall intensity and relative ion intensity. The signal intensity was high, as evidenced by short gating ion times of ∼ 20 μs.

**Figure 2:**
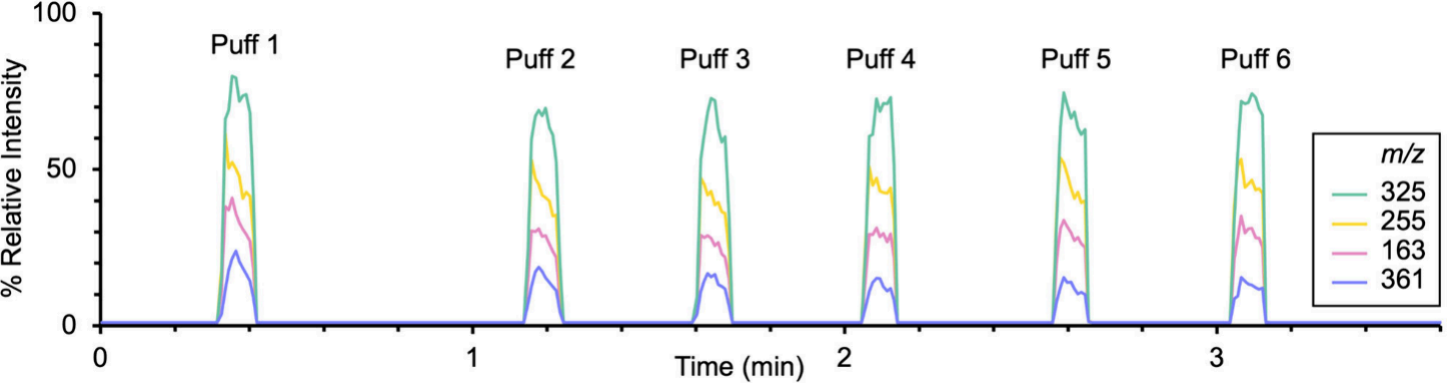
Overlaid extracted ion chromatograms of prevalent positive ions in 5 s aerosol puffs over time from e-cig 1 via mass spectrometry without a conventional ion source. Collected in positive ion mode on a ThermoFisher Scientific LCQ Deca XP Plus MS. The plots were offset from the *x*-axis by 1% relative abundance for baseline clarity.

The left column of Figure 3 contains mass spectra of aerosol puffs from each nicotine device without a conventional ion source. Because mass spectrometers detect only ions, the generation of a signal (or lack thereof) indicated whether the emissions were ionized. Emissions from all four e-cigs were found to be ionized, producing spectral intensities in the range of 10^2^–10^3^ counts (Figure 3c, e, g, and i). In comparison, emissions from the t-cig mouthpiece (Figure 3k) produced low intensity noise of only 7 counts, indicating a negligible ionization signal.

**Figure 3:**
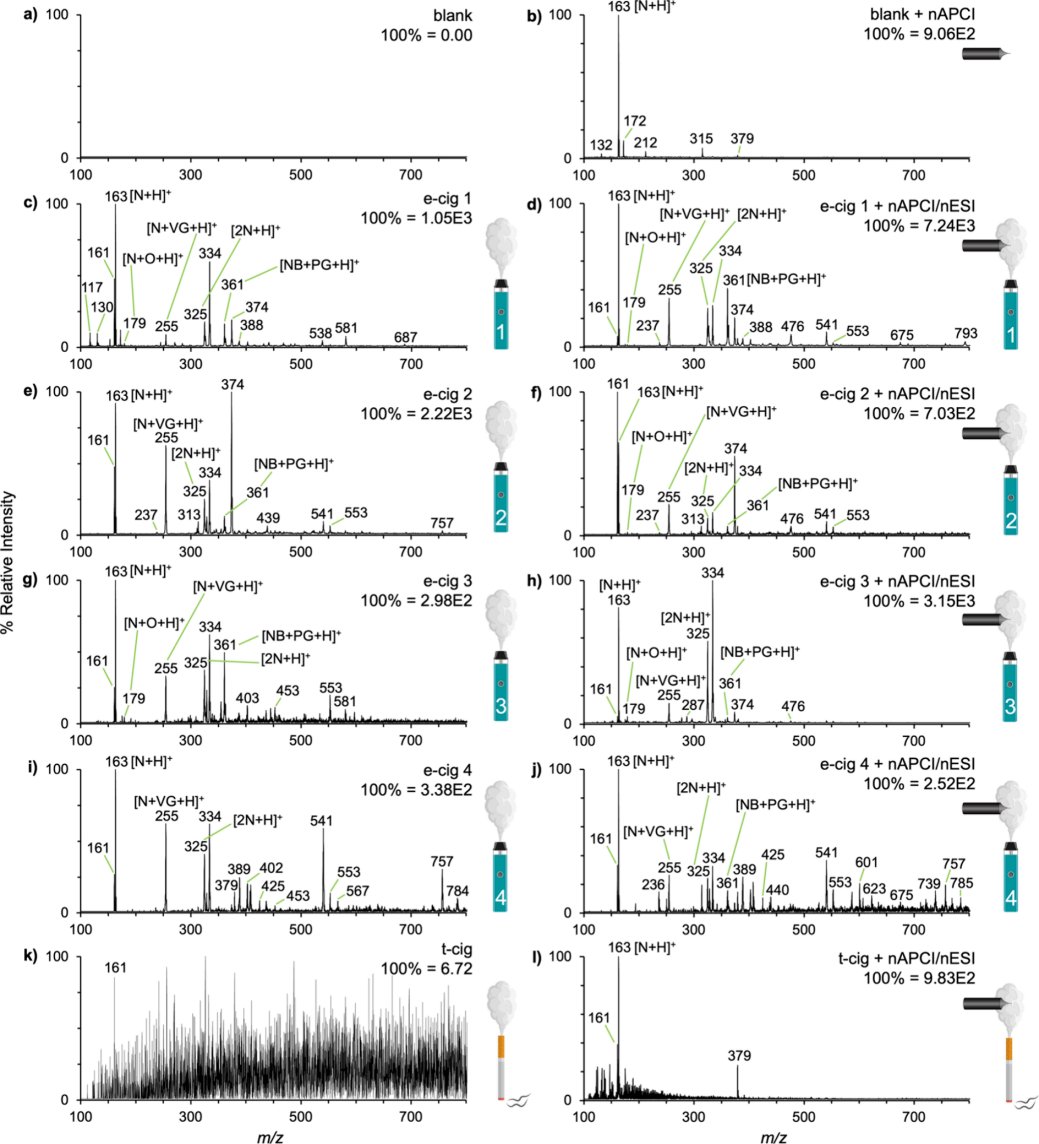
Mass spectra of emissions from each e-cig and t-cig without any conventional ion source (left column) and with the additional nAPCI/nESI source (right column). Includes (a, b) background lab air (blank), (c, d) e-cig 1, (e, f) e-cig 2, (g, h) e-cig 3, (i, j) e-cig 4, and (k, l) a t-cig. All spectra were collected in positive ion mode on a ThermoFisher Scientific LTQ-XL MS and the plots were offset from the *x*-axis by 1% relative intensity for baseline clarity. Provisional peak identifications are included. *N* = nicotine, NB = nicotine benzoate, VG = vegetable glycerin, and PG = propylene glycol.

A 1961 study by Lorenz reported air ion density over time in an enclosed space while a t-cig was smoked ∼ 2 m from a Wesix Ion Collector.^28^ Estimated from the published ion density curve, ion density originating from the t-cig, including unfiltered smoke from the burning tip, peaked at ∼ 1.6 × 10^3^ ions/cm^3^.^28^ This ion density is marginally greater than typical atmospheric background level of 3 × 10^2^ to 1 × 10^3^ ions/cm^3^.^29,30^ While not a direct comparison, mass spectrum intensities in the present study suggest that the ion density from e-cigs was closer to that produced by corona discharge, which is up to 1 × 10^13^ ions/cm^3^ at the needle tip and closer to 5 × 10^6^ ions/cm^3^ after traversing a ∼ 0.75 m flow tube.^31,32^ The effective per-puff inhalation exposure associated with this range of ion densities was not studied and will depend on factors, including respiratory deposition efficiency, gas phase ion chemistry in the respiratory tract, aerosol dilution through simultaneous nasal inhalation, and individual lung capacity and breathing patterns.

The right column of Figure 3 contains mass spectra of aerosol puffs from each nicotine device with the addition of an nAPCI/nESI source. For the e-cigs (Figure 3d, f, h, and j), the presence of the additional ion source did not make a consistent difference in overall signal intensity but did result in different relative intensities of particular ions. Since puff-to-puff variation was shown to be minimal in Figure 2, this change in relative intensity of particular ions may be due to the different ionization mechanisms of e-cig and nAPCI/nESI, ionization occurring at different locations, neutral molecules in the aerosol becoming ionized by nAPCI/nESI, ion annihilation in the corona discharge region, or a combination of these.

For t-cig emissions, exposure to nAPCI/nESI resulted in a full-intensity spectrum (983 counts, Figure 3l) as opposed to low-intensity noise (7 counts, Figure 3k) generated without a conventional ion source. nAPCI/nESI ionized the chemical species in the t-cig smoke, making them detectable by MS. It is known that t-cig smoke contains ions, as all combustion produces ions at some level, but combustion only occurs at the burning tip of the t-cig.^28,29^ Ions formed at the burning tip must traverse the length of the cigarette before leaving the mouthpiece and entering the respiratory tract. Some ion neutralization would be expected along the way, as the ions come in contact with pieces of tobacco leaves, paper, and a filter. E-cigs typically have fewer surfaces with which ions can collide and do not incorporate mouthpiece filters.

When the mixture of PG, VG, nicotine salts, organic acids, and flavorants in an e-liquid is atomized into an aerosol, a variety of oxidation products, thermal degradation products, fragments, adducts, dimers, and complexes can be formed—presumably contributing to the large number and variety of observed ion peaks. All of the ions discussed up to this point have been positive ions, but to a lesser extent all four e-cigs also emitted negative ions, as shown in Figure S1. The negative ion observed from all four e-cigs at *m*/*z* 213 has been assigned to the cluster ion of benzoate and vegetable glycerin, [B+VG]^−^. T-cig emissions produced no signal in the negative mode. All provisionally identified ions observed in Figure 3 are summarized in Table S1.

### E-Cigarette Ionization Mechanisms

An intriguing physical phenomenon of e-cig use is the large, dense aerosol plume of microdroplets formed from the atomization process. Given this work, the plume must contain ions. The droplets are on the order of 2 μm in diameter. Aerosols are known to be highly chemically reactive due to their large surface-to-volume ratio and surface enrichment of ionic species, providing an ideal environment for heterogeneous interfacial reactions. E-cig aerosol droplets are presumably primarily made up of the constituents of e-liquids used in this study, including VG, PG, and nicotine. Glycerin, such as VG, has a high surface tension and has been shown to increase the average charge state of proteins under ESI conditions in a process known as supercharging.^33^ Glycerin has also been used to enhance the ionization process in fast atom bombardment (FAB) ionization, commonly generating glycerin-glycerin and glycerin-analyte cluster ions in the interfacial region of a glycerin matrix.^34,35^ While the observed e-cig self-ionization process is not FAB or ESI, VG and PG may play a role in the ionization process occurring. A PG/VG-assisted ionization mechanism is supported by provisionally identified cluster ions [NB+PG+H]^+^ and [N+VG+H]^+^.

The dominant ionization process in e-cigs appears to be consistent with atmospheric pressure thermospray ionization (APTSI). Classical thermospray, proposed by Blakley and Vestal in 1983, is an MS ionization process in which a solution that typically contains sample and an ionizing buffer is vaporized from a hot tube into a region at low pressure (∼3 Torr), generating charged aerosol droplets.^36,37^ These droplets contain buffer ions and analyte molecules which undergo ion–molecule reactions, yielding analyte ions.^36^ Analyte ions may also exist in solution before vaporization and can be ejected into the gas phase as the aerosol droplets are reduced in size due to evaporation.^38^

Ionizing buffer salts, such as ammonium acetate, are typically added to the sample solution to assist thermospray ionization. No buffers were added to the e-liquids analyzed in this work, but disposable e-cig liquids are typically formulated with nicotine salts for the purpose of reducing throat-burning sensations and increasing the speed of nicotine “buzz” sensation.^39^ Nicotine salt e-liquids contain free-base nicotine (p*K*_a_ 3.12 and 8.02) and an organic acid (typically benzoic acid) to form protonated nicotine in solution, with the proton on the pyrrolidine nitrogen.^39,40^ A protonated nicotine benzoate (NB) and PG cluster ion, [NB+PG+H]^+^ was provisionally identified at *m*/*z* 361 in the spectra from all four e-cigs. Other nicotine salt ion adducts were observed as well, further suggesting a thermospray-like mechanism with nicotine salts acting as an ionization-assisting buffer. The aforementioned PG/VG-assisted ionization process may contribute to or enhance this thermospray-like mechanism.

Although traditional thermospray ionization is conducted at low pressure conditions, APTSI operates at atmospheric pressure and can include configurations incorporating a corona discharge needle similar to the control experiments.^38,41^ Pressure drop across an e-cig reportedly ranges between ∼ 1.5–12 Torr so they are essentially at atmospheric pressure during use, depending on air flow orifice size.^42^ Because of this close alignment with reported APTSI operating conditions, we refer to e-cig self-ionization as an atmospheric pressure process.

Keski-Rahkonen and co-workers reported their APTSI temperature was on the order of 300 °C, which is consistent with typical operating temperatures of heated e-cig filaments under normal use conditions (<330 °C).^38,43^ While the heated atomization filament in an e-cig does not exceed ∼ 330 °C under typical use, this is hot enough for atmospheric pressure thermal desorption ionization (APTDI) in the absence of solvent and presence of a suitable salt, as noted by Cooks and co-workers.^44^ These conditions could be met in the case of a “dry hit” in which all of the liquid in contact with the filament is vaporized and solid nicotine salt residues are left on the filament. Under extreme dry hit conditions, the filament could reach a red hot 1000 °C, which is hot enough for classic thermal ionization.^17,43^ Under such conditions, ion populations would be expected to differ markedly from those produced via softer ionization processes, with differences including analyte degradation, radical cation production, diminished survival of weakly bound clusters, and altered ion distributions.

The presence of a VG/PG plume of droplets along with the predominance of protonated nicotine and intact nicotine-related adducts observed in spectra therefore suggest that both forms of thermal ionization are unlikely to be the dominant pathway under typical e-cig operating conditions, though they may contribute to a greater extent during atypical, less frequent high-temperature scenarios such as dry hits, which were not investigated. The analytical observations reported here are most consistent with APTSI assisted by nicotine salts and PG/VG as depicted in Figure 4, however, multiple ionization pathways may contribute depending on e-liquid formulation, device design, and the operating conditions of an individual device over time.

**Figure 4:**
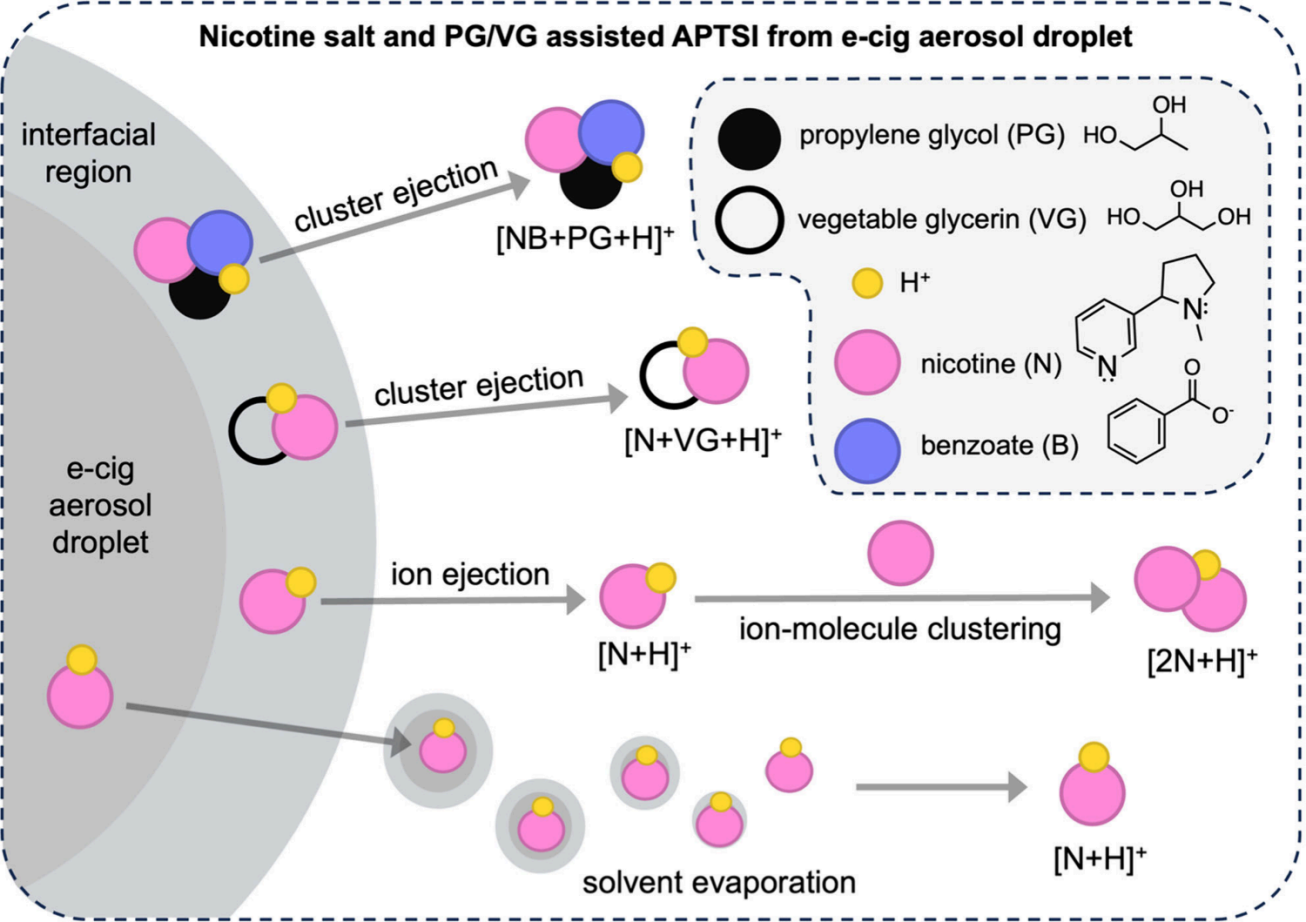
Diagram of proposed e-cig self-APTSI schemes under typical operating conditions at the air interface of an e-liquid aerosol droplet, showing how various assigned ions could have been formed. Partially adapted from Székely and Allison’s FAB work.^35^ *N* = nicotine, NB = nicotine benzoate, VG = vegetable glycerin, PG = propylene glycol.

### Physiological Impacts of Inhaling Concentrated Gas Phase Ions

The high abundance of gas phase ions in e-cig aerosol warrants consideration as a distinct exposure pathway when health risks are evaluated for e-cig users. To place this observation in context, the following discussion synthesizes relevant findings from the existing literature on ambient air ion exposure. Gas phase ions are also generated by natural and anthropogenic sources such as cosmic radiation, waterfalls, crashing waves, lightning, dust storms, combustion, high voltage power lines, and traffic exhaust, which contribute to ambient air ion density.^29,30,45,46^ The health impacts of ambient ion exposure have been debated for over a century.^47,48^ Some studies report that exposure can induce physiological effects.^28,47,49,50^ Other studies indicate that ambient level exposure has no health impacts due to low ion density and low probability of systemic absorption.^29,48^ At high ion density, however, there is more evidence to suggest that inhalation is correlated to physiological changes.

Ions behave differently than neutral species, favoring deposition on respiratory surfaces rather than being exhaled.^30,51–54^ This is because their deposition is primarily dependent on electrostatic forces rather than aerodynamics or diffusion.^51,54–56^ This mechanism dominates in the smaller, deeper airways like the alveoli, which is where respiratory gas exchange occurs.^56,57^ Ion deposition could intensify chemical disruption of the lung lining surfactant layer, contributing to alveolar collapse and increasing absorption into respiratory tissue.^12^ These processes may lead to a higher effective dose than would be estimated based on neutral particles alone and may help explain the rapid onset effect of nicotine salt e-liquids if they facilitate ion formation in APTSI.

Ion inhalation has been correlated with impaired respiratory function. Cilia, the respiratory system’s first defense against infection, move mucous containing trapped pathogens and debris out of the body via nose or mouth.^49^ Exposure to 1 × 10^6^ negative or positive gas phase ions/cm^2^ generated by beta radiation disrupted ciliary motion and mucus production in small mammal respiratory tissue, with effects persisting after exposure.^49^ Control experiments suggested an oxidation-driven mechanism. In human children and adults, exposure to 5–10 × 10^5^ positive air ions/cm^3^ was associated with respiratory congestion and aggravated exercise-induced bronchial response, likely due to impaired ciliary function.^47,58^ Prolonged exposure to high concentrations of positive gas phase ions increased susceptibility to infection in mice, indicating immune suppression consistent with chronic e-cig use.^47,59^

Gas phase ion inhalation has also been shown to disrupt levels of serotonin, an important neurotransmitter, in small mammals and humans. Negative ions reduced free serotonin levels and increased the levels of its oxidation product in small mammals, suggesting an oxidative pathway.^47^ Conversely, positive ions elevated blood serotonin levels in small mammals.^60^ Both negative and positive ions reduced brain serotonin in small mammals after prolonged exposure.^61^

Densely ionized air has exhibited bactericidal effects, disrupting microflora on solid media and aqueous solutions.^47,50,62^ Gas phase ion jets have been applied clinically in dentistry for sterilization purposes.^63^ Under typical circumstances, however, the oral microbiome plays a key role in maintaining oral health and preventing infection.^16^ E-cig use has been correlated with oral microbiome disruption and impaired antioxidant activity in saliva, suggesting oxidative pathways.^16^

Concentrated gas phase ion inhalation also has been correlated with systemic effects including altered growth rate, skin temperature, pulse, and reaction time.^28^ Small mammals exposed to negative air ions at 3 × 10^5^ ions/cm^3^ for 60 min exhibited elevated ROS markers.^64^ Air ions from coal burning power plants also triggered high ROS levels and cellular injury, implicating oxidative mechanisms.^71^ Findings across multiple systems indicate that concentrated gas phase ion exposure can alter biological function, although substantial uncertainty remains regarding mechanistic pathways. While these studies provide important context for interpreting the potential implications of ionized e-cig emissions, direct evaluation of biological responses to inhaled e-cig ions remains an important area for future toxicological investigation. Further research is also needed to identify and deconvolute contributions of individual ions, ion-facilitated oxidative processes, and other chemical interactions, which could vary based on ion type and exposure conditions.

### Direct Molecular Analysis of E-Cig Aerosol Without a Conventional Ion Source

An abundant ion produced by all four e-cigs was *m*/*z* = 255. Forbes and Krauss previously identified this ion as a proton bound cluster of nicotine and VG, [N+VG+H]^+^.^7^ Figure 5 contains spectra focused on *m*/*z* 255 from background air and each nicotine device analyzed, zoomed in from Figure 3. The broad ∼ 1 Da full width at half-maximum (fwhm) of the *m*/*z* 255 peak is indicative of a weakly bound cluster or adduct undergoing metastable dissociation upon ejection from a linear ion trap due to collisions with the helium damping gas at mTorr pressure. This behavior further supports the peak assignment, as metastable dissociation leading to peak broadening is expected for weakly and noncovalently bound clusters such as [N+VG+H]^+^ under these conditions.

**Figure 5:**
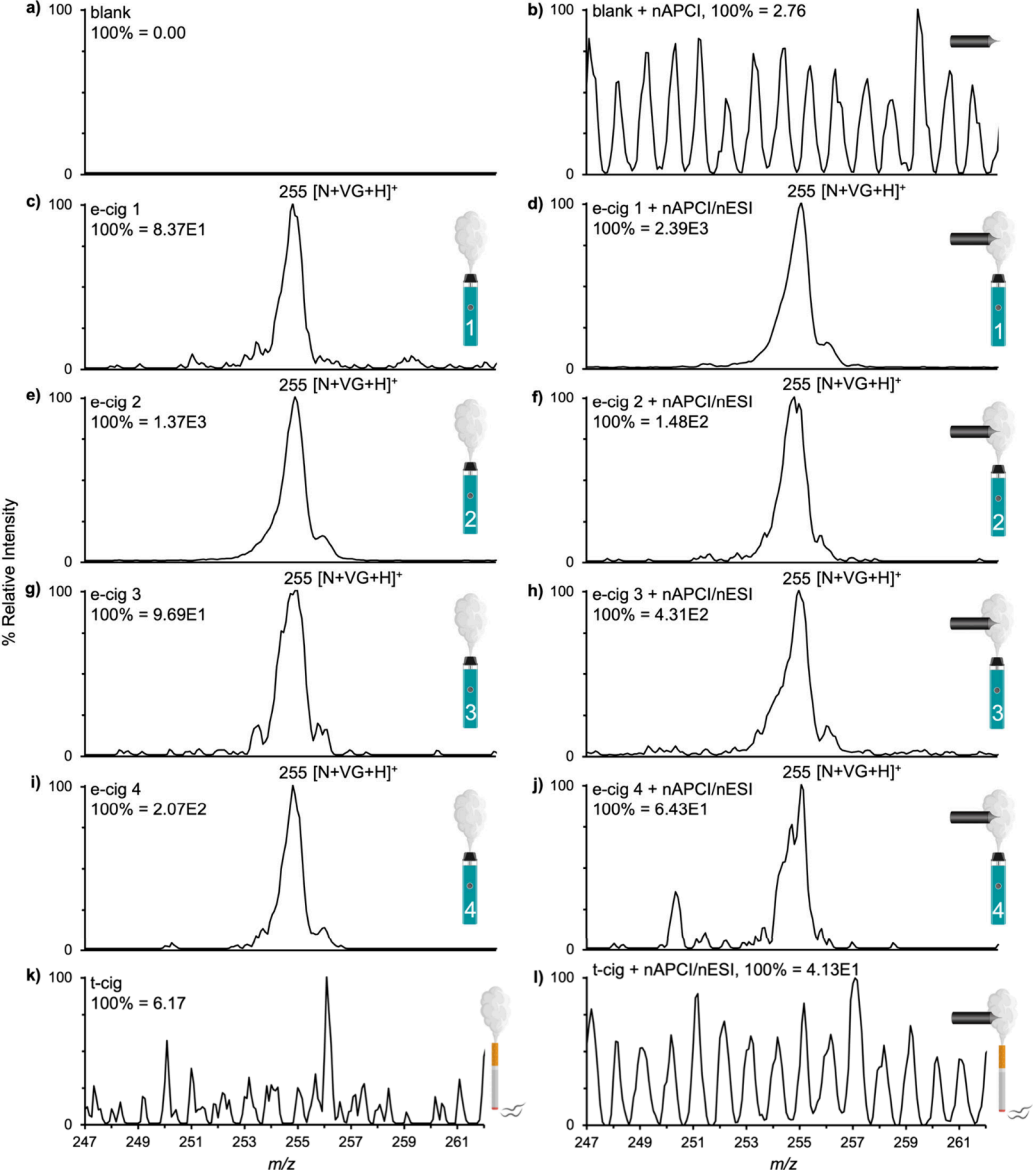
Mass spectra over the *m*/*z* range 247–262 of emissions from each e-cig and t-cig without any conventional ion source (left column) and with an nAPCI/nESI ion source (right column). Includes (a, b) background lab air (blank), (c, d) e-cig 1, (e, f) e-cig 2, (g, h) e-cig 3, (i, j) e-cig 4, and (k, l) a t-cig. All spectra were collected in positive ion mode on a ThermoFisher Scientific LTQ-XL MS and the plots were offset from the *x*-axis by 1% relative intensity for baseline clarity. Provisional peak identifications are included. *N* = nicotine, VG = vegetable glycerin.

The charged cluster was directly observed in emissions from all four e-cigs without a conventional ion source (Figure 5c, e, g, and i). Adding the nAPCI/nESI ion source changed the peak intensities (Figure 5d, f, h, and j). In contrast, t-cig emissions without an ion source produced only low-level noise with irregular peak shapes due to negligible ion abundance (Figure 5k). When t-cig emissions were ionized by nAPCI/nESI, the resulting spectrum had a higher intensity and more typical peak shapes (Figure 5l), closely resembling the blank background air ionized by nAPCI (Figure 5b). Despite sufficient ion generation (Figure 3l), no [N+VG+H]^+^ cluster was observed at *m*/*z* 255, consistent with VG not being a major constituent of t-cig tobacco.

Protonated nicotine, [N+H]^+^, at *m*/*z* 163 was another prominent ion detected.^7^ Figure 6 presents the mass spectra from Figure 3 zoomed in to the nicotine region for each device and background laboratory air. Emissions from all four e-cigs contained [N+H]^+^ without the use of a conventional ion source (Figure 6c, e, g, and i). When nAPCI/nESI was activated, the [N+H]^+^ signal intensities changed (Figure 6d, f, h, and j). In contrast, t-cig emissions without an ion source produced only low-level noise with irregular peak shapes due to a low signal (Figure 6k). Ionization of t-cig emissions by nAPCI/nESI yielded ∼ 100 times higher intensity at *m*/*z* 163, with smooth peak shapes throughout the mass spectrum (Figure 6l).

**Figure 6:**
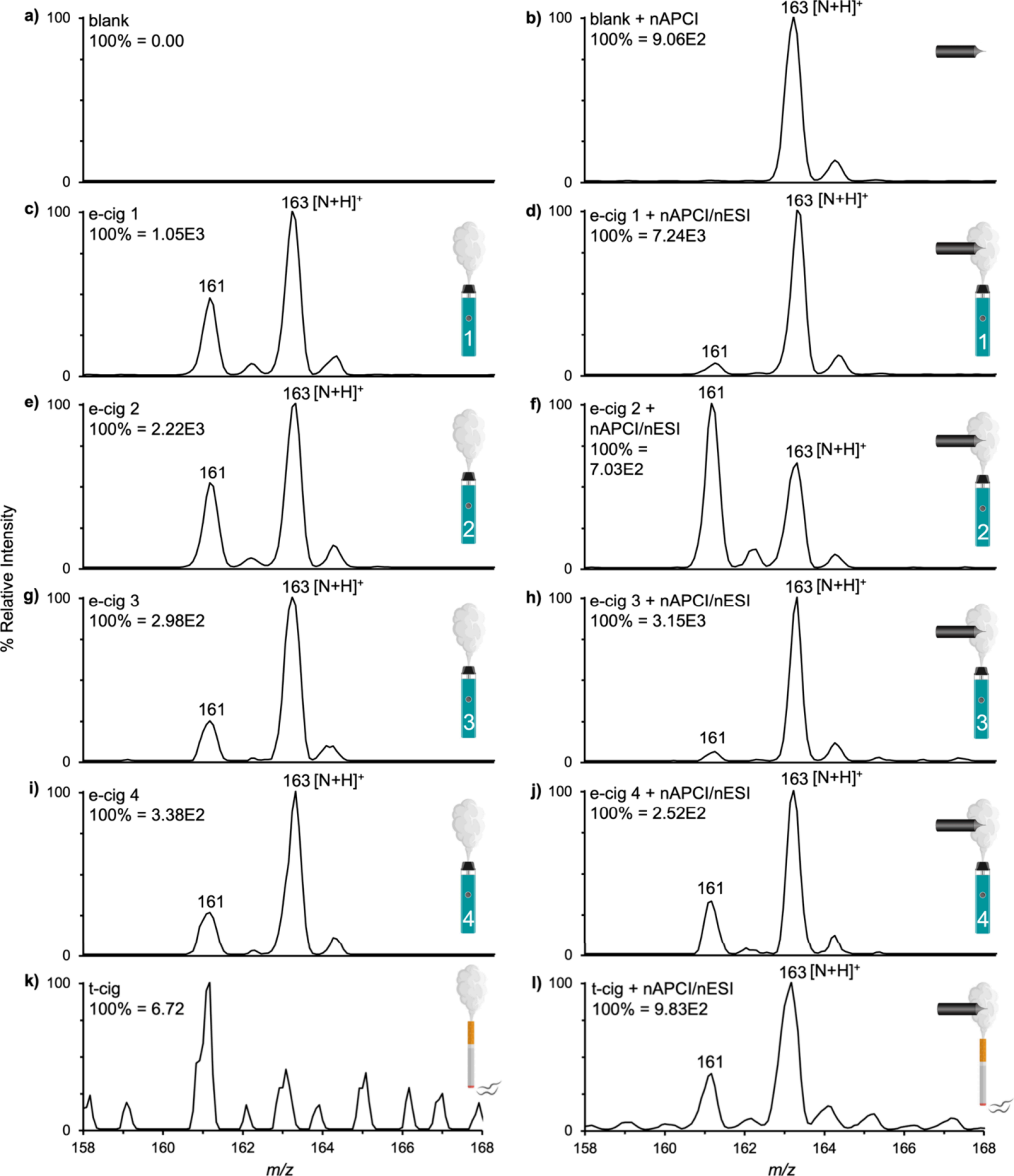
Mass spectra over the *m*/*z* range 158–168 of emissions from each e-cig and t-cig without any conventional ion source (left column) and with an nAPCI/ESI ion source (right column). Includes (a, b) background lab air (blank), (c, d) e-cig 1, (e, f) e-cig 2, (g, h) e-cig 3, (i, j) e-cig 4, and (k, l) a t-cig. All spectra were collected in positive ion mode on a ThermoFisher Scientific LTQ-XL MS instrument and offset from the *x*-axis by 1% relative intensity for baseline clarity. The *m*/*z* 163 is assigned to protonated *N* = nicotine.

The blank spectrum in Figure 6b notably contains a [N+H]^+^ peak. This is attributed to gas phase nicotine from e-cigs in the off state, low level ambient nicotine in the room air from earlier runs, and surface residue left over from previous experiments in the plumbing being ionized by nAPCI and contaminating the background spectrum. An advantage of using e-cigs as their own ion producing source was the lack of background ion signals (Figures 6a, 4a, and 5a). Numerous ions, including provisionally identified [N+H]^+^ and [N+VG+H]^+^, were detected in real time directly from e-cig emissions without a conventional ion source, whereas nAPCI was required to generate a signal from smoke pulled out of the t-cig by the pump.

Nicotine metabolism in humans starts with enzyme-catalyzed 5′ oxidation to form an iminium ion,^65^ which is subsequently oxidized into cotinine by aldehyde oxidase, generating superoxide and associated oxidative lung damage.^66^ Peaks at *m*/*z* 161 observed in all e-cig emissions (Figure 6, left column) are consistent with the presence of nicotine-derived iminium ions. Such species could form via gas phase oxidation by hydroxyl radicals (OH^•^) present in e-cig aerosols,^14^ which can abstract hydrogen from the 2’ or 5′ carbon of nicotine to generate an organic radical that is further oxidized to nicotine-Δ1′(5′)-iminium and nicotine-Δ1′(2′)-iminium ions (C_10_H_13_N_2_^+^, *m*/*z* 161, Figure 7).^40,67^ The reproducible observation of *m*/*z* 161 across all e-cig devices, together with its mass agreement and consistency with established nicotine oxidation pathways, supports this assignment. Additionally, protonated nicotine was observed in the blank spectrum (Figure 6b), while *m*/*z* 161 was not. This supports the proposed mechanism since, in the absence of an activated e-cig, radicals would not be present to initiate the reaction.

**Figure 7:**
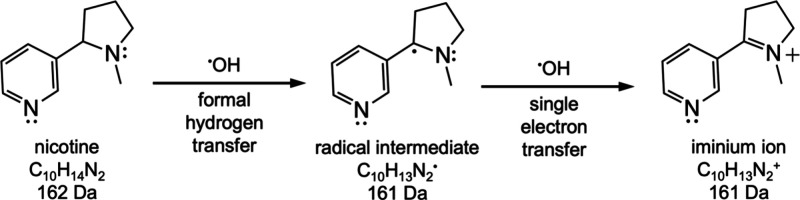
Potential ^•^OH radical dehydrogenation reaction for the formation of the iminium ion, [C_10_H_13_N_2_]^+^, from nicotine. Nicotine-Δ1′(2′)-iminium ion is depicted, but its isomer nicotine-Δ1′(5′)-iminium could also be readily formed through this oxidative pathway.^40^

The direct emission of such electrophilic ions from e-cigs could introduce these biologically reactive species directly to the lung surface. Ionized organic molecules may also interact with OH^•^ and biomolecules in the lung lining fluid to form other ROS.^68,69^ The analytical observations presented here, together with established nicotine oxidation mechanisms, provide a plausible physiochemical context for ROS-related chemistry but do not constitute direct evidence of ROS formation in biological systems following e-cig aerosol inhalation.

Adding an nAPCI/nESI source to e-cig emissions did not appreciably affect the *m*/*z* 161 counts, indicating that corona discharge or electrospray ionization did not significantly contribute to the formation of this ion (Figure 6). In contrast, for t-cig emissions, *m*/*z* 161 counts increased substantially from 7 (Figure 6k) to 372 (Figure 6l) upon nAPCI/nESI ionization, which may be attributed to ionization of anatabine, a tobacco alkaloid with protonated *m*/*z* of 161 previously detected directly from t-cig smoke, rather than iminium ion formation.^70^

Gas phase organic ion dimerization reactions have been reported to be induced by conventional APCI ion sources, confounding accurate determination of the sample’s original composition.^21^ Evidence of induced reactions in the form of differing ionization mechanisms can be seen in the incongruent relative abundances of the spectra collected with, versus without, a conventional ion source. Allowing the e-cig to act as its own ionizer bypasses the use of conventional ion sources, eliminating issues like this as well as the need for background subtraction and resulting in spectra inherently representative of the ion mixture entering the respiratory tract.

## Conclusion

The analytical observations reported here provide evidence that e-cigarette aerosols can directly emit concentrated gas phase ions under typical operating conditions. While multiple ionization pathways may contribute, a prominent pathway appeared to be APTSI assisted by nicotine salts and PG/VG. There are two major implications of e-cig self-generating ions. First, a more direct and accurate atmospheric pressure MS analysis of the e-cig aerosol is made possible by allowing the e-cig to act as its own ion source, eliminating confounding ions from conventional ion sources. Second, inhalation of concentrated gas phase ions from e-cigs may be physiologically harmful due to their chemical and electrostatic properties. The ion-dense nature of e-cig aerosol adds another layer of complexity to our understanding of e-cigs and may help explain the observed health symptoms associated with e-cig use. Further studies are needed to determine the contributions and nature of the different ionization mechanisms, the nature of the glycerin/glycol droplets as it relates to ion formation, the identities of more ions, the pervasiveness of this phenomenon among e-cig devices, and the mechanism of gas phase ion inhalation toxicity.

## Supplementary Material



## Data Availability

The MS data acquired in this work are publicly available via the Carnegie Mellon University KiltHub repository at DOI: 10.1184/R1/29828978.

## Notes

Negative mode mass spectra of e-cig emissions, t-cig emissions, and a blank without a conventional ion source; table of provisional ion identifications (PDF)
