# Supplementary material for: E‑Cigarettes Generate Gas Phase Ions
Source: Environ Health (Wash). 2026 Apr 20;4(7):1556–66. doi: 10.1021/envhealth.5c00788 (PMC13386374; doi:10.1021/envhealth.5c00788)
Supplement: Supplementary file 1 [file eh5c00788_si_001.pdf]

Supporting Information

E-Cigarettes Generate Gas Phase Ions

Nicole C. Auvil[1] and Mark E. Bier*[1]
[1]Department of Chemistry, Carnegie Mellon University, 4400 Fifth Avenue, Pittsburgh, Pennsylvania 15213, USA
*Correspondence: mbier@cmu.edu

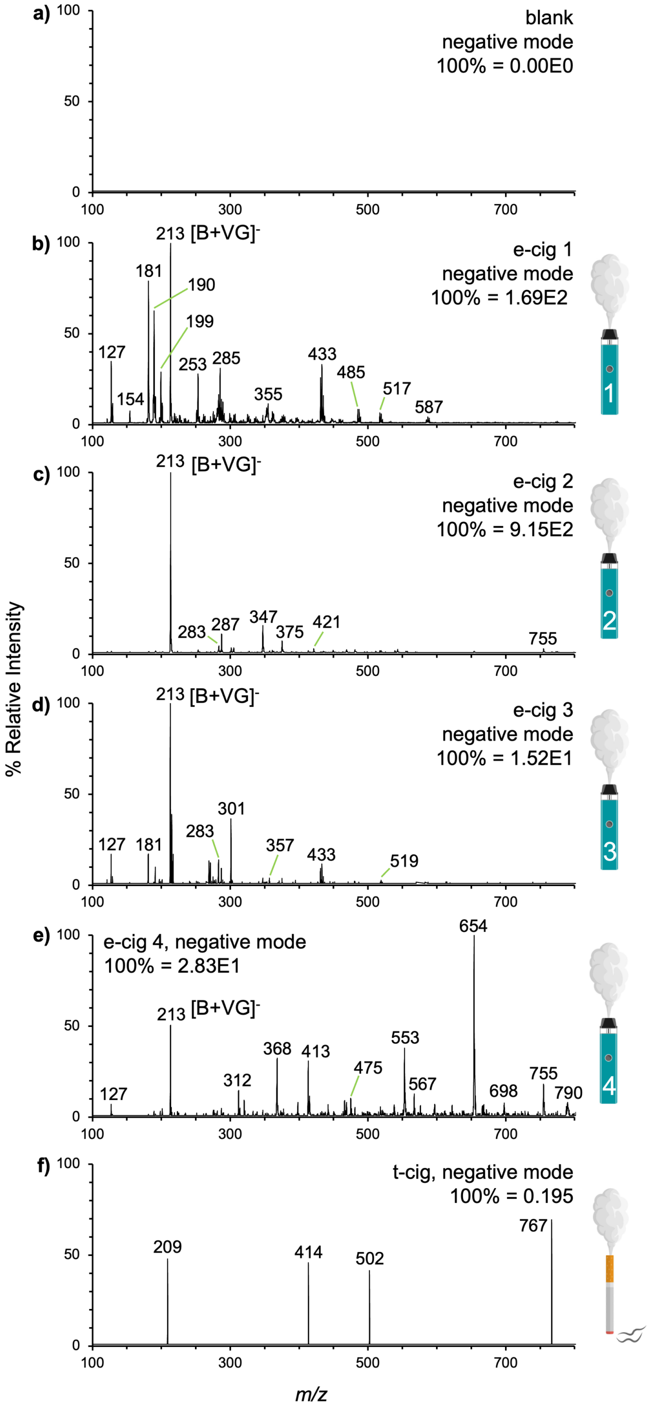

Figure S1. Negative mode mass spectra of emissions from a) background lab air (blank), b) e-cig 1, c) e-cig 2, d) e-cig 3, e) e-cig 4, and f) a t-cig. All were collected with no conventional ion source. Each spectrum was background subtracted, normalized to the base peak, and offset from the x-axis by 1% relative intensity for baseline clarity. Collected on a Thermo Fisher Scientific LTQ-XL MS. Peak widths in the t-cig spectrum indicate that these peaks are instrumental artifacts. Provisional peak identifications are included. B= benzoate, VG= vegetable glycerin

Observed *m/z*	Proposed Assignment	Supporting Observations/Limitations
161 (+)	nicotine-Δ1′(5′)-iminium and/or nicotine-Δ1′(2′)-iminium	Assignment based on known nicotine oxidation pathways and nominal mass agreement; cannot distinguish between potential isomers
163 (+)	$[Nicotine+H]^+$	Assignment based on prior work
179 (+)	$[Nicotine+O+H]^+$	Assignment based on known oxidation during atomization and nominal mass agreement
255 (+)	$[Nicotine+VG+H]^+$	Assignment based on prior work; broad peak consistent with metastable complex
325 (+)	$[2Nicotine+H]^+$	Assignment based on prior work
361 (+)	$[Nicotine\ Benzoate+PG+H]^+$	Assignment based on nominal mass agreement and consistency with typical e-liquid nicotine salt compositions
213 (-)	$[Benzoate+VG]^-$	Assignment based on nominal mass agreement and consistency with typical e-liquid nicotine salt compositions

Table S1. Summary of provisionally identified gas phase ions observed in e-cig emission mass spectra, including *m/z* values, proposed assignments, and supporting observations/limitations.
